# Classic and modern models of self-regulated learning: integrative and componential analysis

**DOI:** 10.3389/fpsyg.2024.1307574

**Published:** 2024-03-07

**Authors:** Carolina Tinajero, Mª Emma Mayo, Eva Villar, Zeltia Martínez-López

**Affiliations:** Department of Developmental and Educational Psychology, University of Santiago de Compostela, Santiago de Compostela, Spain

**Keywords:** components, phases, models, review, self-regulated learning

## Abstract

Self-regulated learning (SRL) is considered a construct of great heuristic value and has attracted the attention of numerous researchers and inspired influential theoretical models. The objective of the present study was to provide an up-to-date, comparative and integrated description of the theoretical models of SRL used in current empirical research. For this purpose, we conducted a critical review of the scientific literature referring explicitly to any SRL model and we described, compared and integrated the processes and personal and situational dimensions considered in each model. The models have clearly evolved from focusing on cold self-regulation, conscious activity and individual functioning, to emphasising hot self-regulation and considering implicit activity and interindividual functioning. Among empirical research lines based on the most recent models, the following stand out: detailed analysis of SRL during its progress, the manifestation of SRL in diverse instructional formats and the role of affective/motivational self-regulation.

## 1 Introduction

Self-regulated learning (SRL) has been defined as an active constructive process, in which students’ thoughts, feelings and actions are self-generated and deliberately oriented to achieving personal learning goals, and which is influenced by environmental factors (Boekaerts, 1996a,b; Pintrich, 2000; Zimmerman, 2000). Students use different types of learning strategies (e.g., cognitive, motivational, etc.) that they select, execute and adapt according to their aims and depending on their personal dispositions and characteristics. It is a situated process, in which various distant (e.g., family educational patterns and school climate) and proximal (e.g., instructions and resources regarding a task in process) contextual factors play a key role, thus determining the acquisition and implementation of learning strategies (Ben-Eliyahu and Bernacki, 2015).

The historical origin of the SRL construct is usually considered to have occurred in 1986, when the *American Educational Research Association* organized a symposium with the aim of combining contributions of particular importance regarding what were then judged to be the essential components of strategic functioning in the educational field: learning strategies, metacognition, self-concept, volitional strategies and self-control (Zimmerman, 2008). Numerous research studies have since been conducted in relation to SRL, yielding a multitude of articles and monographs (see, e.g., Boekaerts et al., 2000; Schunk and Green, 2018). These reports aim to clarify the nature and development of SRL, its relationship with academic achievement and the role of personal goals in self-regulation processes, among other aspects. The importance that SRL has acquired is demonstrated by the attention given to the construct in current Educational Psychology handbooks and by the international recognition of the value of self-regulation as a basic skill that should be promoted in educational systems (Ananiadou and Claro, 2009).

The construct has undoubtedly been of great heuristic value, inspiring global theoretical models in which phases and components (processes and personal and situational dimensions) are delimited. The proposed SRL models, from the earliest to the most recent, differ in aspects such as their theoretical background and the detail and emphasis with which the SRL phases and components are treated. Critical reviews by Panadero (2017) and Puustinen and Pulkkinen (2001) have helped to reveal common points and differences in the various models. By taking these reviews into consideration and adopting a similar approach to analyze the most recent theoretical proposals, the main aim of this study was to provide an up-to-date, comparative and integrated description of the main SRL models, i.e., those referred to in the scientific literature as valuable for exploring the nature of the SRL components and their interrelationships and conditioning factors. More precisely, we aimed to undertake the following:

- Compile the main theoretical models of SRL that guide empirical research on the construct, including graphical representations.
- Describe the main assumptions of the models and the essential characteristics of the representations, highlighting the contributions of each.
- Disentangle the SRL components (processes and personal and situational dimensions) considered in each model and compile a comprehensive, integrated list of these components.

This critical review addresses the following research questions:

RQ1: What are the essential characteristics and components of existing theoretical models on SRL?

RQ2: How have theoretical models of SRL evolved?

## 2 Method

We conducted a literature search in the WOS and PsycInfo databases, using the expression “self-regulated learning AND model*,” for the period from 2015 to the present. In order to encompass the diversity of theoretical developments and empirical lines of research inspired by SRL models, we decided to prioritize the sensitivity of the search strategy over its specificity. Thus, we selected generic search terms. The search expression used was “self-regulated learning AND model*.” In total, we compiled 705 references, 11 of which were duplicates. The reports retrieved consisted of 688 peer reviewed articles and 6 book chapters.

We examined the theoretical basis of the reports retrieved, selecting those that explicitly referred to an SRL model or review of SRL models (total, 198). Exclusion criteria were not applied. The complete text of each study selected was screened for theoretical background and citations of theoretical models of SRL. Finally, we compiled the original publications reporting each of the models referred to in the reports reviewed and proceeded to summarise them. Focus was placed on the components and processes of the models as well as on the assumptions about their interrelations.

## 3 Results

The number of reports published, expressly based on SRL models, has increased gradually, with 2021 and 2022 being the most productive years. Most of the reports selected (51%) were explicitly based on the cyclical phase model developed by Zimmerman (1998b, 2000). The model of Winne and Hadwin (1998) was the second most cited (35%), followed by those of Pintrich (2000) (31%) and Boekaerts (2006, 2007) (12.6%). Unsurprisingly, given that there will be a time lag before the publication of relevant research findings in relation to the theoretical proposals, the most recent models were the least commonly cited. In addition, the review article by Panadero (2017) was cited in 16.6% of the reports, while that by Puustinen and Pulkkinen (2001) was less frequently cited (5%).

Below we present a summarised description of the characteristics and central assumptions of the SRL models selected. For this purpose, we have included figures representing the models and we have focused on emphasising the characteristics of each in the text. Thus, the reader can observe the evolution and status of SRL construct, which have been shaped by the most outstanding authors in the field.

### 3.1 Classic models

We consider the models included in this section as classic, as they have served to forge a body of assumptions shared by different psychological perspectives, which constituted an important stimulus for the research on SRL (Zeidner et al., 2000; Usher and Schunk, 2018).

The proposals of Zimmerman (1989, 1990, 1994, 1998a,b, 2000) are some of the first and most widely recognised in relation to SRL. Specifically, in the Triadic Analysis of SRL, Zimmerman (1989, 1990) adopts the assumptions of the social cognitive perspective of human self-regulation and proposes multidimensionality as its essential characteristic. This model considers three sources of reciprocal influence involved in self-regulation and that should be considered in the field of education: personal (covert beliefs, such as self-perception and knowledge of one’s own regulatory processes, and affective processes), behavioural (covert and overt conduct) and environmental (physical and social context). Projection of these sources of influence on the agentic functioning of students led (Zimmerman, 1994, 1998a) to distinguish different dimensions of self-regulation related to fundamental research questions: motivational (*why* is the individual taking part in the learning process), methodological (*how* the individual approaches the learning process), temporal (*when* the different steps of the personal action plan are applied), behavioural (*what* overt conduct is initiated/modified), contextual (*where* the learning takes place, in terms of the physical environment) and social (*who* the student can and would like to count on for support during the learning process).

**Figure 1:**
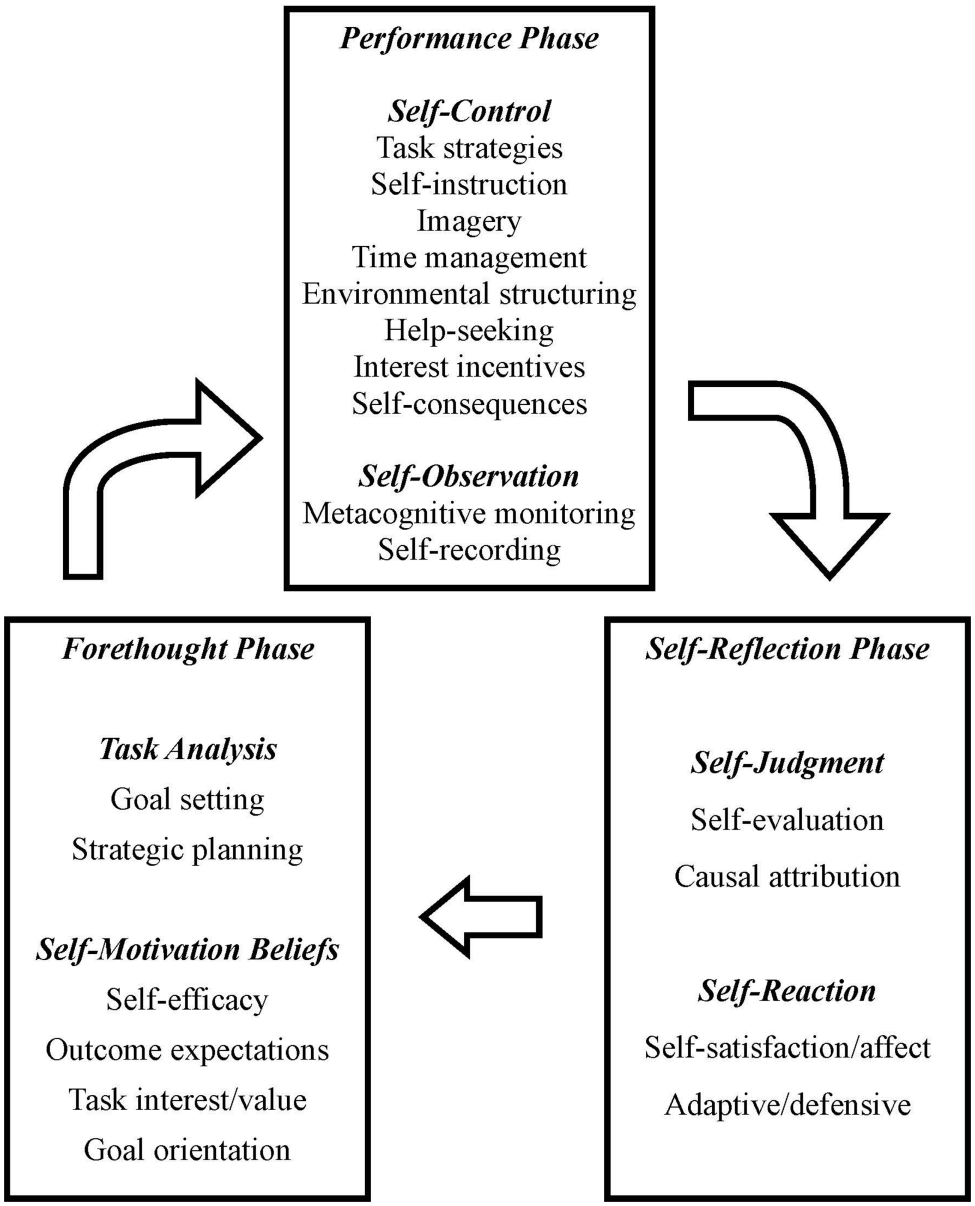
Zimmerman and Moylan's cyclical model. From Zimmerman and Moylan (2009, p. 300). Copyright (© 2009) and Imprint. Reproduced by permission of Taylor & Francis Group.

Finally, the cyclical phase model (Zimmerman, 1998b; Zimmerman and Moylan, 2009) delimits three recursive stages of SRL (see Figure 1):

1. Forethought, which includes processes that precede and form the basis of the learning effort and the development of the self-regulation process, particularly to establish objectives.^1^
2. Performance, related to the processes that take place during the learning task and that affect attention and the course of action.
3. Self-reflection, which involves processes posterior to task execution, and accounts for cognitive and motivational reactions in response to the learning experience and that form the basis of the forethought phase in subsequent trials of the learning cycle.

**Figure 2:**
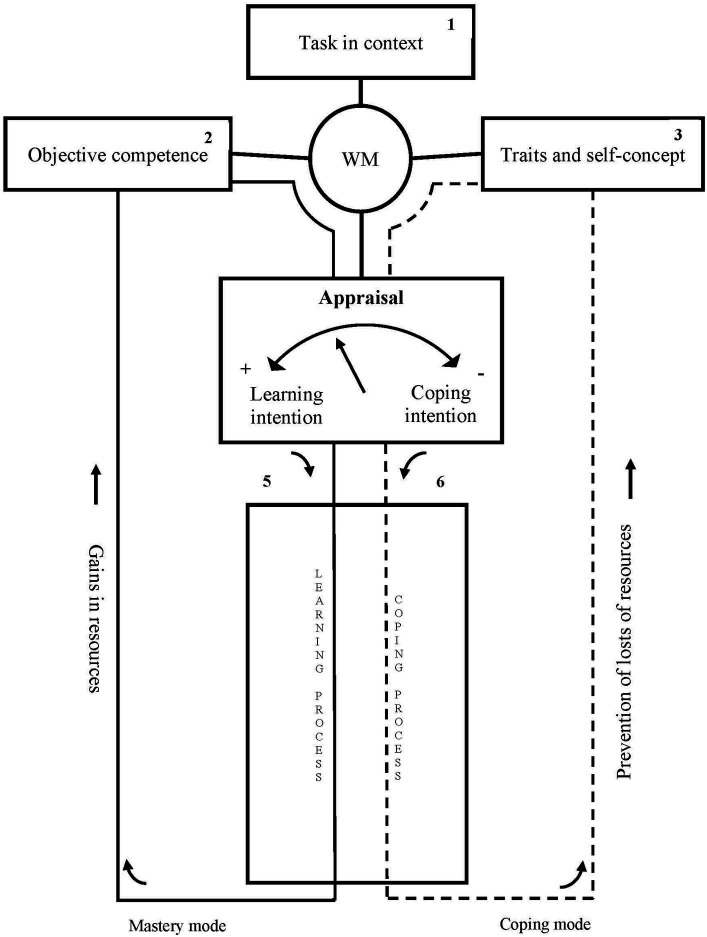
Boekaerts' adaptable learning model. WM = working model. From Boekaerts (1996a, p. 456). Copyright (1996) by John Wiley & Sons, Inc. Reproduced with permission.

The models developed by Boekaerts (1991, 1995, 2006, 2007) have also had an important influence in the field of educational psychology. This author focuses on the role of the motivational dynamics that drive the individual within the SRL cycle. Her adaptable learning model (see Figure 2) considers two alternative processing modes (mastery and coping), which correspond to the preponderance of one or other type of the principal motives of the student when confronted with a learning task.

The mastery mode originates in the aspiration to expand the personal repertoire of knowledge and skills and entails activation of learning strategies. On the other hand, the coping mode is brought about by the desire to preserve well-being and prevent any possible loss, damage or distortion of this state (Boekaerts, 1991, 1995) and involves activation of self-defence strategies, which may hamper learning. The balance between these modes depends on the *appraisal* based on an internal model of the learning situation (*working model*, WM), conformed according to three sources of information: (1) the characteristics of the task in question (demands and conditions in which it is presented); (2) the domain, declarative and procedural information that the student possess relative to the task, and (3) the contents of the self-system which are activated by the task (motivational values and beliefs). The appraisal may involve the perceived congruence between the value attributed to and the resources available for conducting the task, which will produce positive affective reactions and direct the student towards the mastery mode. On the other hand, appreciation of incongruencies, which may threaten personal well-being, will generate negative affective states and direct the individual towards the coping mode. The individual’s actions linked to a task may be initiated by either of these routes and then change depending on successive appraisals of the task being undertaken.

**Figure 3:**
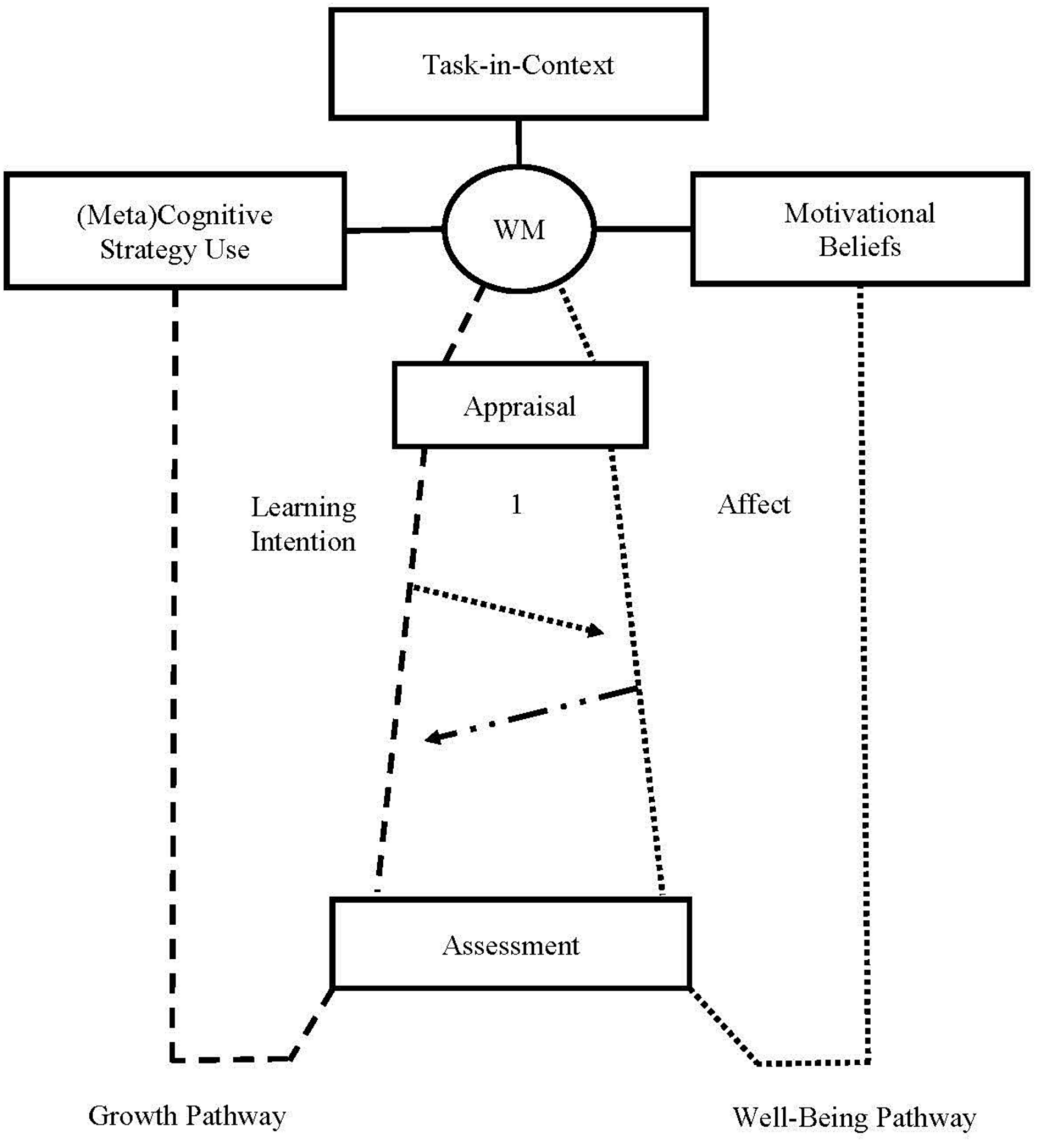
Boekaerts´dual processing model. WM = working model. From Boekaerts (2006, p. 350). Copyright (2006) by John Wiley & Sons, Inc. Reproduced with permission.

After the initial formulation of her model, Boekaerts showed increasing interest in the circumstances that determine the transition between the two alternative routes of processing and the role of the satisfaction of basic psychological needs and also in volitional processes that maintain or, where applicable, return the student to the mastery mode (see, e.g., Boekaerts and Niemivirta, 2000). The author further explored theoretical arguments in support of her view of SRL, compiled empirical evidence and finally presented her a dual-processing model (Boekaerts, 2006, 2007) (see Figure 3). This model includes a volitional self-regulation path, which ranges from the coping pathway (now denominated well-being) to the mastery pathway (now growth) and which reflects the student’s attempts to remain focused on the task, despite any obstacles or distractions that may arise. This would involve the use of different strategies aimed at controlling affective and motivational reactions associated with the difficulties found in undertaking the task.

**Figure 4:**
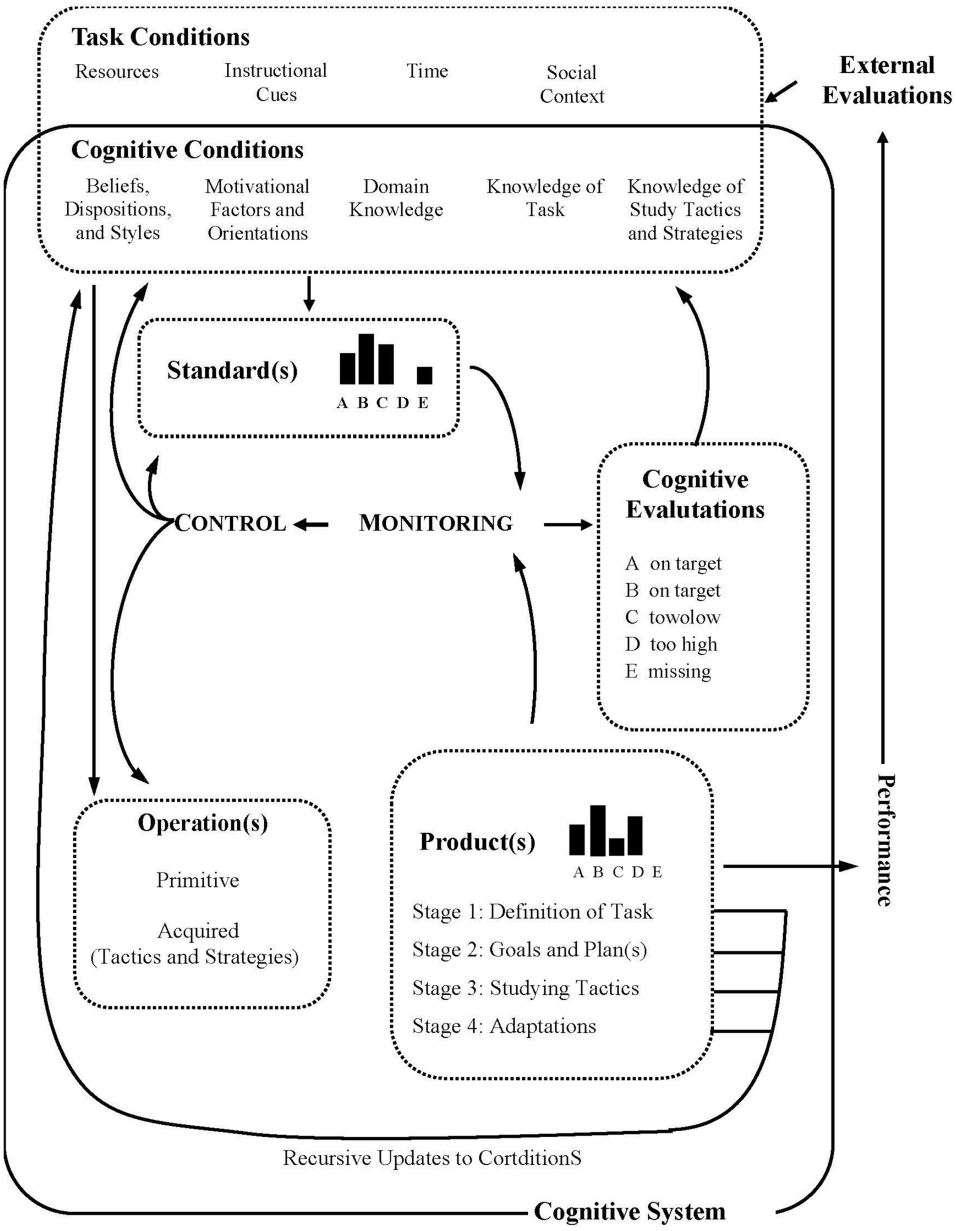
Winne and Hadwin's COPES metacognitive monitoring and control. From Winne and Hadwin (1998, p. 329). Copyright (© 1998) and Imprint. Reproduced by permission of Taylor & Francis Group.

Coinciding with publication of the previous models, Winne and Hadwin (1998) also made an influential proposal, elaborated from the perspective of information processing. In the proposal, the student’s monitoring of their own cognitive activity acquires a central role, as a key process that guides control of the activity in each of the phases of SRL (see Figure 4). Illustrative examples of the way in which monitoring is manifested in the different phases of SRL are given in the original release of the model and in later publications by Winne and colleagues (Winne and Perry, 2000; Winne, 2001, 2011; Winne and Hadwin, 2008).

The planning phase identified in Zimmermann’s model (Zimmerman, 1998b, 2000) is divided into (1) task definition and (2) goal setting and planning. The available information about the task conditions, including the specifications provided for execution (e.g., materials and knowledge required, individual or group learning method, etc.), is processed in the first phase. These appear in Figure 4 as standard (s). The student can decide to monitor this initial representation of the task, ensuring correct understanding of the demands. In the second phase, the student generates a personal profile of selected standards, i.e., a set of objectives in terms of ideal, optimal or satisfactory states to achieve, regarding behaviour, cognition or motivation. Once this profile has been established, the operations (tactics and strategies) that constitute the plan of action are activated. The plan can be monitored metacognitively (mentally tested), which may lead to redefinition of the task or of the plan of action. In the third phase (enacting tactics), the student carries out the plan, the results of which are also monitored and, in this case, checked against the personalized standards. This internal feedback will be complemented by any external feedback provided and can also lead to changes in the profile of standards and the plan of action. Optionally, on completing the task, in a fourth (adaptation) phase, the student monitors the overall way that the procedure has occurred, generating an improved representation of the task features and the best way of acting, which can later be applied to similar tasks.

The phases are implicitly included in the diagram of the model (Figure 4), in which a series of boxes that include essential components of SRL stand out. We will consider these components in the following section.

Finally, any review of the classic models must include mention of those developed by Pintrich (2000, 2004), who, also inspired by a social cognitive view, integrated both phases and areas of self-regulation, which are, respectively, placed in the columns and rows of a representative table (see Table 1). The model subdivides the performance phase into two (monitoring and control) and subsumes the 6 dimensions of self-regulation differentiated by Zimmerman in four areas (cognition, motivation/affect, behaviour and context). Pintrich also adds specifications about the personal processes and dimensions involved, based on metacognitive and motivational notions.

**Table 1:** Pintrich’s phases and areas model.

	Areas for regulation			
Phases	Cognition	Motivation/affect	Behaviour	Context
1 Forethought, planning and activation	Target goal setting	Goal orientation adoption	[Time and effort planning]	[Perceptions of task]
	Prior content knowledge activation	Efficacy judgments	[Planning for self-observations of behavior]	[Perceptions of context]
	Metacognitive knowledge activation	Ease of learning judgments: perceptions of task difficulty Task value activation Interest activation		
2 Monitoring	Metacognitive awareness and monitoring of cognition (feelings of knowing, judgments of learning)	Awareness and monitoring of motivation and affect	Awareness and monitoring of effort, time use, need for help	Monitoring changing task and context conditions
			Awareness and monitoring of effort, time use, need for help Self-observation of behavior	
3 Control	Selection and adaptation of cognitive strategies for leaning, thinking	Selection and adaptation of strategies for managing motivation and affect	Increase/decrease effort	Change or renegotiate task
			Persist, give up Help-seeking behavior	Change or leave context
4 Reaction and reflection	Cognitive judgments Attributions	Affective reactions Attributions	Choice behavior	Evaluation of task Evaluation of context

Brackets indicate reference to cognitive-volitional processes. From Pintrich (2000, p. 454). Copyright (2000) by Elsevier Inc. Reproduced with permission.

Regarding the metacognitive facet, in the first phase of SRL (forethought, planning and activation) the model of Pintrich (2000) includes *efficacy judgements* and *ease of learning judgements*, derived from the knowledge about the task and its context and self-knowledge in relation to the task (in the table, metacognitive knowledge). The monitoring phase includes *feelings of knowing* and *judgements of learning*, the latter being related to the fluidity of task processing and impediments that arise during its execution.

Regarding the motivational facet, the model includes self-efficacy, attributions, task value and affective reactions; the activation, monitoring and control of these are explicitly contemplated in the different phases of the model. The inclusion of self-regulation of motivational dimensions is, in fact, one of the distinctive features of the model. The so-called *goal orientations* deserve special mention for the special role that the author confers them in SRL. Thus, the author conceives these as general motives explaining why the student engages in academic tasks, which originate from the representations of desired results and/or states, and which condition the monitoring and control processes used during task execution (Pintrich, 2000).

Indeed, Pintrich’s interest in both the (meta) cognitive and motivational facets of learning is patent in studies published prior to the model, and it is projected in the *Motivated Strategies for Learning Questionnaire* (MSLQ; Pintrich et al., 1991), which is probably the questionnaire most widely used internationally to evaluate SRL. Pintrich’s model has been given visibility by its integrative nature as it explicitly includes cognitive, motivational, behavioural, and contextual dimensions and thus highlights the multidimensionality of SRL (Limón et al., 2004).

### 3.2 Modern models

In general, the models presented below can be characterized as derivations of the classic models in four directions: from a macro (generic) to a micro (detailed/situated) focus; from cold to hot self-regulation (by the gradual weighting of affective-motivational states and their self-regulation); from conscious to implicit activity; and from individual to interindividual functioning.

**Figure 5:**
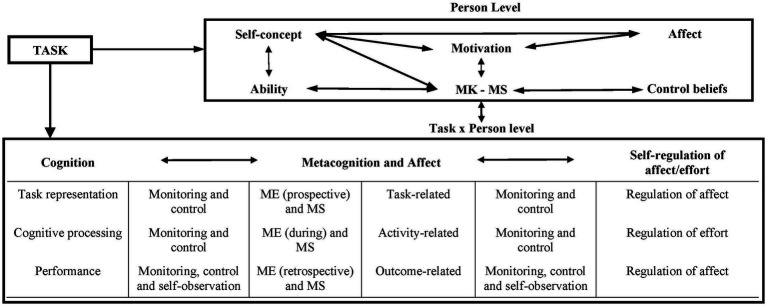
Efklides’ metacognitive and affective model of self-regulated learning. metacognitive knowledge; MS = metacognitive skills; ME = metacognitive experiences. From Efklides (2011, p. 7). Copyright © by Division 15, American Psychological Association. Reproduced by permission of Informa UK Limited, trading as Taylor & Francis Group, www.tandfonline.com on behalf of Division 15, American Psychological Association.

The *Metacognitive and Affective Model of Self-Regulated Learning* (MASRL) developed by Efklides (2011, 2018) is a clear example of the first three tendencies. This model distinguishes two levels of processing, which include three types of metacognitive phenomena differentiated by Flavell (1979) (see Figure 5). Thus, the macro or *Person* level, comprising stable characteristics of the individual that transcend specific learning episodes, includes *metacognitive knowledge*. This refers to declarative information about oneself, academic tasks and learning strategies stored in memory and that form the basis of performance of academic tasks. The micro or *Task x Person* level includes online processing of the task, in which *metacognitive experiences* become important. These experiences include judgements and feelings generated during task monitoring, in the three phases of SRL contemplated in the model (task representation, cognitive processing and task performance) and the active knowledge related to the task. Finally, *metacognitive skills* condense procedural knowledge (represented in the macro level) and the practical application (represented at the micro level) of strategies for controlling cognition (executive processes), understood as conscious and intentionally displayed procedures.

The author considers in detail the role of metacognitive experiences, regarded as manifestation of metacognition in everyday situations, and she emphasizes the role of *metacognitive feelings* (e.g., confidence in carrying out a task correctly or satisfaction with having achieved an established objective). These feelings emerge unconsciously and transmit the personal relevance attributed to a particular learning task, endowing the cognitive act with affective load (pleasant or unpleasant emotions) associated with the cognitive act. The author of the model also contemplates the possibility that unconscious heuristic processes, i.e., routines established by experience with other similar tasks, also participate in SRL processes. On the other hand, Efklides (2011) draws attention to the role of metacognitive experiences in the social shaping of cognition, as well as in teaching SRL and in collaborative learning dynamics, aspects which this author studied prior to publication of the MASRL model (Salonen et al., 2005; Efklides, 2008).

**Figure 6:**
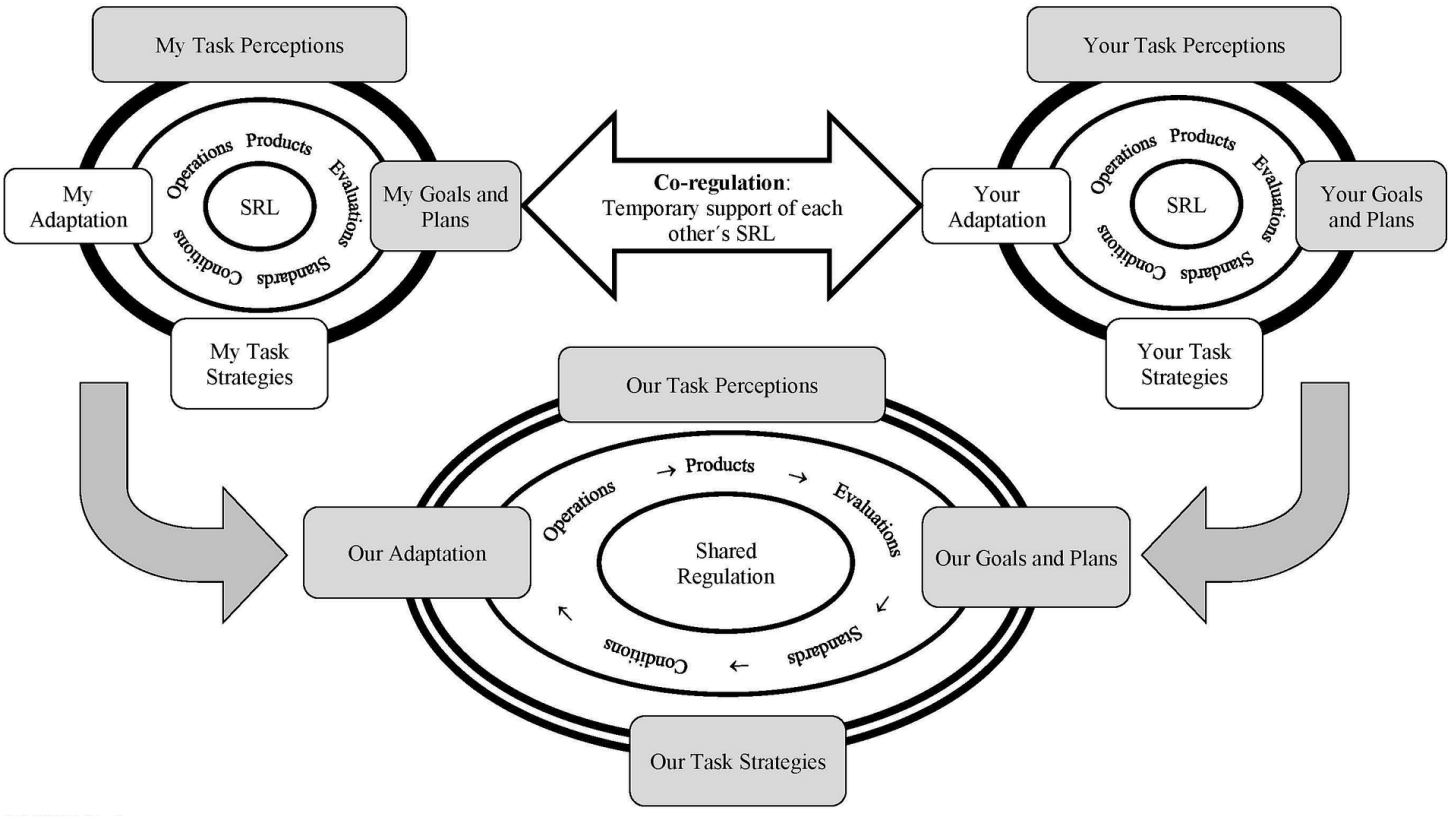
Hadwin et al.'s model of socially shared regulated learning. From Järvelä and Hadwin (2013, p. 29). Copyright © Division 15, American Psychological Association. Reproduced by permission of Informa UK Limited, trading as Taylor & Francis Group, www.tandfonline.com on behalf of Division 15, American Psychological Association.

The model developed by Hadwin et al. (2011), Järvelä and Hadwin (2013), and Hadwin et al. (2018) illustrates precisely the necessary social nature of SRL and differentiates three modes in which this can be manifested, in an interactive and collaborative learning environment (see Figure 6).

First, *self-regulated learning*, which refers to the functioning of each student separately, regarding the same task. The authors emphasize that, even in this case the SRL process is a socio-historic and environmentally situated process, in the sense that it is shaped by personal and group beliefs and experiences, by the context of the task and by the involvement, along with others, in its execution. Second, *co-regulated learning,* consisting of the stimulation produced by the self-regulated learning experience of another, giving rise to exchange or internalization of self-regulation processes. Third, *socially shared regulation*, produced when the self-regulation processes are interdependent and/or jointly constructed during episodes of cooperative learning.

**Figure 7:**
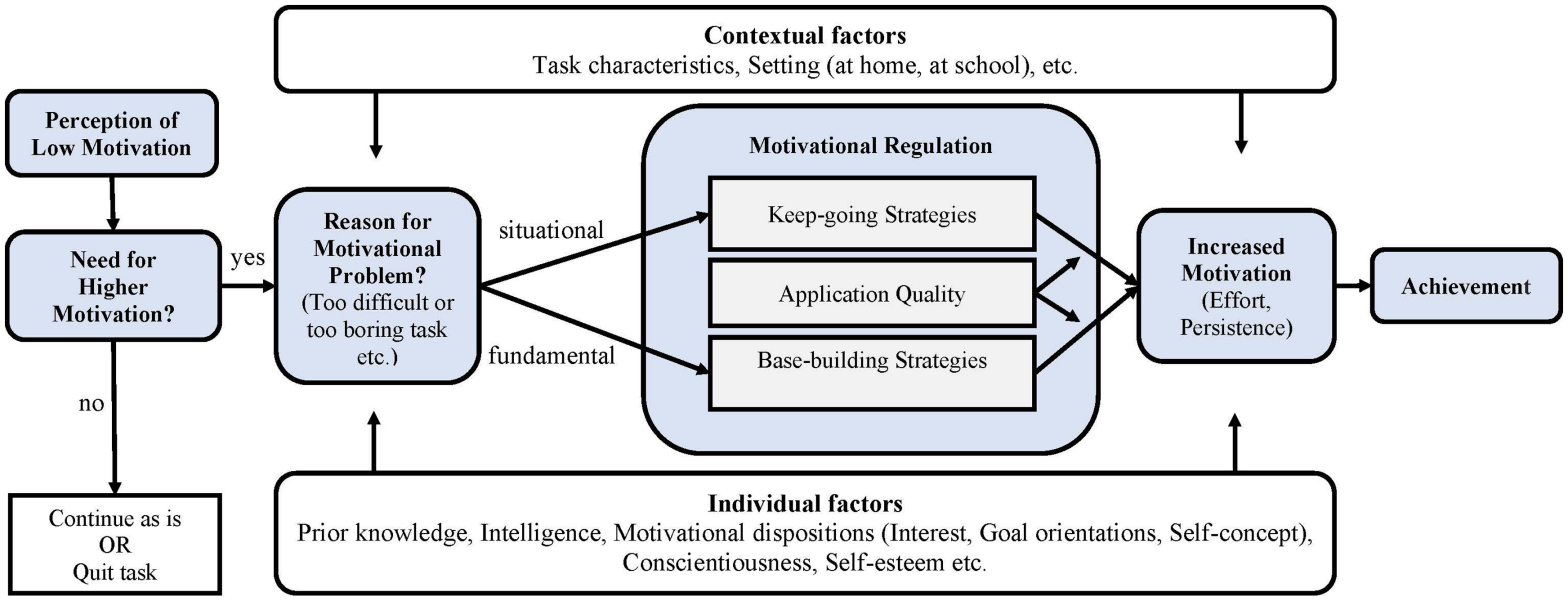
Schwinger and Stiensmeier-Pelster's model of motivational regulation. From Schwinger and Stiensmeier-Pelster (2012). Copyright (2012) by Elsevier Inc. Reproduced with permission.

We consider the motivational regulation model of Schwinger and Stiensmeier-Pelster (2012) a clear example of the gradual incorporation of hot aspects of self-regulation. This model directs our attention towards the role of the strategies that the students use to regulate their motivation, as an essential factor determining their performance. Such strategies are activated in response to the realization that the motivational state is insufficient for continuing with a task once initiated (see Figure 7).

Once the deficit has been perceived, the student deduces the cause, which may be *situational* (transitory) or *fundamental* (stable). Taking these aspects into account, the student will select which strategies of motivational self-regulation to apply and the way of doing so (either maintaining the activity or elevating the basic motivation). The efficacy of the process is assumed to depend on the student’s skill in detecting a possible deficit in motivation and adjusting the strategy accordingly. Regarding the latter, the contribution made by Schwinger’s group can be considered essential, i.e., design of the *Motivational Regulation Questionnaire* (MRQ; Schwinger et al., 2009), a tool that has been well received by the scientific community.

**Figure 8:**
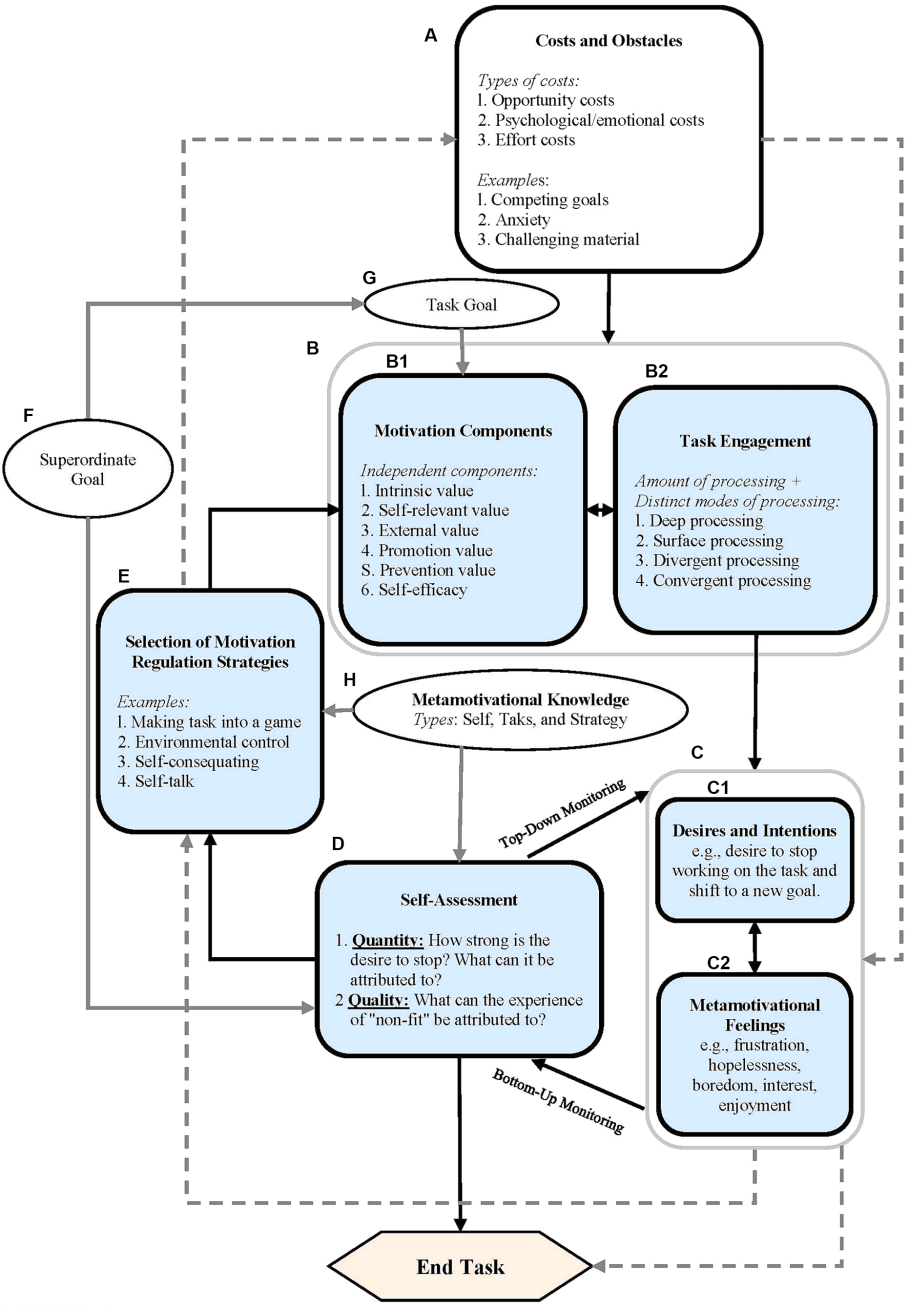
Miele and Scholer’s model of motivational regulation. From Miele and Scholer (2018, p. 2). Copyright (© 2017) by Division 15, American Psychological Association. Reproduced by permission of Informa UK Limited, trading as Taylor & Francis Group, on behalf of Division 15, American Psychological Association.

Miele and Scholer (2018) provide a more recent, detailed conceptualization of motivational self-regulation in their Metamotivational Model (see Figure 8).

In this model, the initial motivation to engage in a task is understood to be formed as a function of a specifically established objective (oval G in Figure 8; e.g., getting a good mark in an exam), which in turn depends on some type of aspiration of a higher order (oval F; e.g., performing well throughout the course). The self-efficacy and the task value are specified as motivational dimensions to be monitored and controlled (box B_1_ in Figure 8), at the start and throughout execution of the task. The state of these dimensions is assumed to be bidirectionally associated with the processing mode used in the task (box B_2_); this association is modulated by the predicted cost and obstacles that will occur during execution (box A). Monitoring the motivational state (*metamotivational monitoring*; routes between C and D) can occur in a downwards direction, when it is controlled by executive processes, such as, e.g., when the student evaluates whether their motivation is sufficient to allow a plan of action to be carried out. On the other hand, it can occur in an upwards direction when it is guided by *metamotivational feelings* (phenomenological experiences such as pleasure or frustration), indicative of the state of the motivational components and, when applicable, of the possible risk of abandonment or change in the initial objective. This process is sustained in *metamotivational knowledge* (oval H), i.e., that related to the motivational requisites of the task, the motivational self-regulation strategies and the personal ability to execute the strategies. Finally, the motivational self-regulation strategies play a key role in the *metamotivational control* (route from box E to B), i.e., in maintaining or increasing the level of motivation for carrying out a specific task, with an established objective. The model authors point out that metamotivational monitoring and control can proceed in a conscious or automatic manner.

**Figure 9:**
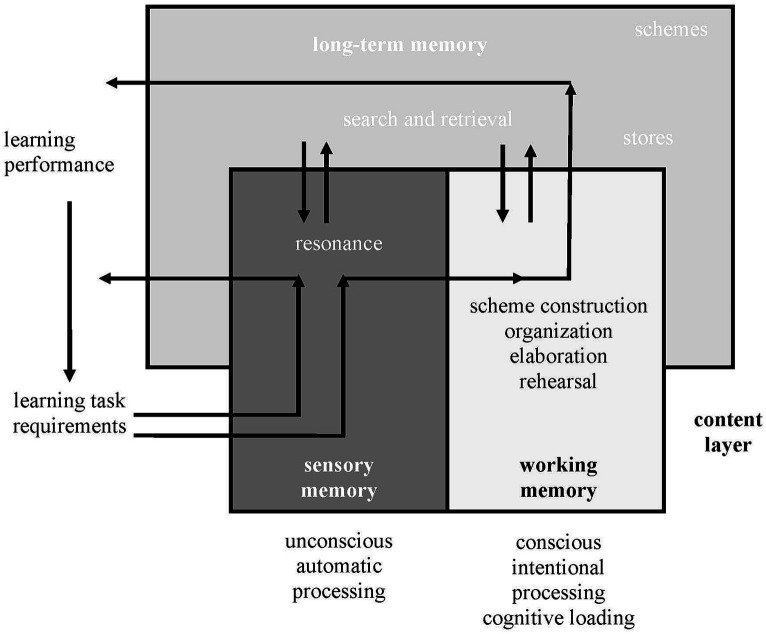
Wirth et al.’s interactive layers model. From Wirth et al. (2020, p. 1132). Copyright (2020) by Springer. CC-BY-NC.

Both of these modes of processing are specifically represented in the interactive layers model recently proposed by Wirth et al. (2020) (Figure 9). The authors include sensorial memory as a necessary explanatory structure, through which information from the environment and that activated by the task in the individual student enters the cognitive system. The information may be of three types (learning content, cognitive procedures and metacognitive procedures) corresponding to three simultaneous layers of processing. The authors provide a representative figure for each of the layers. The figure including the learning content layer is shown below by way of example.

If the information that the sensory memory accesses coincides with that stored in the long-term memory, resonance occurs, i.e., the coincidental information is reinforced and acquires prominence in the learning process, which can proceed unconsciously. However, if the resonance is sufficiently intense or lasting, a conscious process may occur, whereby the resonant information is intentionally processed in the short-term memory. On the other hand, the non-resonant information is discarded, except when it is sufficiently strong, in which case a search is initiated for concordant information in the long-term memory.

A common feature of all of the models described is that they all have a dynamic view of SRL, i.e., they propose a cyclical sequence of events that form a prototypical generic learning episode (Zeidner and Stoeger, 2019). Different interdependent components come into play throughout the sequence (processes, dispositions, states and environmental conditions). As an alternative mode of analyzing the nature of SRL, some authors have considered differentiating and classifying these components, proposing what have come to be known as static or componential models (Wirth and Leutner, 2008; Sitzmann and Ely, 2011), in contrast to the models described so far, distinguished as dynamic. Below we present an analysis of the treatment of SRL components in both types of proposals.

### 3.3 Components analysis

We have compiled five published classifications of SRL components, which we present in Table 2, with the aim of facilitating comparison of the similarities and differences. As can be observed, the first two classifications subdivide the components depending on whether they correspond to motivation or cognition, understood as domains of self-regulation in the approach used by Garcia and Pintrich (1994) and as regulatory systems in that used by Boekaerts (1996b). A coincident transverse organization can also be noted, which corresponds to the facets of metacognition differentiated by Flavell (1979) and specifically recognised in the dynamic models of Pintrich (2000, 2004) and Efklides (2011, 2018): metacognitive knowledge, skills and experiences. However, the last type of component is only included in the last three classifications, giving these a more situated nature than the first two.

**Table 2:** Components of self-regulated learning (SRL) considered in static models.

Garcia and Pintrich (1994)	Boekaerts (1996b)	Winne and Hadwin (1998)	Pintrich et al. (2000)	Efklides (2006, 2008)
Beliefs about task/class: - Goal orientation - Personal interest - Classroom norms Self-schemas: - Affect [self-esteem] - Temporal sign [past, present future selves] - Efficacy - Value/centrality [placed on the task]	Metacognitive knowledge and motivational beliefs [domain specific knowledge related to tasks]: - Beliefs, attitudes, and values - Strategy beliefs - Capacity beliefs - Goal orientation	Conditions: - Interest - Goal orientation - Learning styles - Time constraints - Available resources - Knowledge of tactics - Task knowledge - Subject matter expertise Products: - Task definition - Goals & plans - Tactics enacting - Adaptation Standards: - Ideal, optimal, or satisficing states in relation with the task, objective(s) and plans, studying tactics, and adaptations	Metacognitive knowledge: - Knowledge of cognition and cognitive strategies - Knowledge of tasks and contexts - Knowledge of self	Metacognitive knowledge: Ideas, beliefs, theories of person/self, task, strategies, goals, cognitive functions, validity of knowledge, theory of mind
Conceptual knowledge: - Content knowledge - Disciplinary knowledge Metacognitive knowledge: - Regarding tasks - Regarding strategies	Content domain: - Conceptual knowledge - Procedural knowledge - Misconceptions - Inert knowledge			
Motivational strategies - Self-handicapping - Defensive pessimism - Self-affirmation - Attributional style	Motivational regulatory strategies - Mental representation of behavioral intention - Linking behavioral intention to action plan - Maintaining action plan in the phase of obstacles and competing action tendencies - Disengaging action plan and behavioral intention Motivation strategies - Create learning intention - Coping processes to alter stressors and to reduce negative emotion - Prospective and retrospective attributions - Effort avoidance - Using social resources	Operations: - Searching - Monitoring - Assembling - Rehearsing - Translating	Self-regulation and control: - Planning activities - Strategy selection and use - Allocation of resources - Volitional control	Metacognitive skills: Conscious, deliberate activities and use of strategies for: Effort allocation, time allocation, orientation/monitoring of task requirements/demands, planning, check and regulation of cognitive processing, evaluation of the processing outcome
Regulatory learning strategies - Goal-setting - Planning - Monitoring - Self-testing Cognitive learning strategies - Rehearsal - Elaboration - Organization Results - Effort (quantity and quality) - Self-schema activation/restructuring - Knowledge activation/restructuring - Choice - Persistence - Academic performance	Cognitive regulatory strategies - Mental representation of learning goals - Design of action plan - Monitoring progress and evaluation goal achievement Cognitive strategies - Selective attention - Decoding - Rehearsal - Elaboration - Structuring - Generating questions - Activation of rule(s) + application - Repair: reapply a rule, search for a new rule, decide that no rule is available - Proceduralize a skill			
		Evaluations: Judgments about the task, objective(s) and plans, studying tactics, and adaptations	Metacognitive judgments and monitoring: - Task difficulty or ease of learning judgments - Learning and comprehension monitoring or judgments of learning - Feeling of knowing - Confidence judgments	Metacognitive experiences: Feelings of familiarity, difficulty, knowing, confidence, satisfaction Judgments/estimates: of learning, source memory information, estimate of effort, estimate of time Online task-specific knowledge Task features Procedures employed

The proposal of Winne and Hadwin (1998) deserves special mention. This model includes five componential categories (represented by the acronym COPES), which we understand are also similar to the metacognitive facets. Thus, the category *conditions* groups components that determine the personal representation of the task, including the available resources and restrictions, derived from the external context (e.g., instructional clues and social dynamics of the classroom) and internal conditions (e.g., prior knowledge of the learning strategies and styles); this is therefore the equivalent of metacognitive knowledge. The *operations* category combines the different modes of cognitive manipulation of the information (tactics and strategies) and thus corresponds to metacognitive skills. These generate *products*, a third category that includes the cognitive, motivational, affective and behavioural results of the operations. These products constitute new conditions for successive phases of SRL. In fact, in the dynamic model of Winne and Hadwin (1998), the phases take the names of the characteristic products generated: (1) task definition, (2) goals and plan (s), (3) study tactics and (4) adaptations. The category *evaluations* includes the judgements and feelings generated during execution of the task, thus coinciding with the metacognitive experiences. Finally, the category *standards* combines attributes, in terms of ideal, optimal and satisfactory states, which the student aspires to in the task being executed. These constitute the task objectives and serve as reference points for successive evaluations. In this respect, the standards can be considered part of metacognitive knowledge.

With the aim of evaluating the importance attributed to the SRL components and localizing their position in the cyclical sequence reflected in the dynamic models, we have elaborated a comparative table (Table 3), in which we list the components that explicitly appear in the models.

**Table 3:** Components of self-regulated learning considered in dynamic models.

Zimmerman and Moylan (2009)	1. Forethought		2. Performance		3. Self-reflexion	
	Motivational beliefs: - Self-efficacy - Outcome expectations - Task interest/value - Goal orientation	Task analysis: - Goal setting - Strategic planning	Self-control: - Task strategies - Self-instruction - Imagery - Time management - Environmental structuring - Help seeking - Interest incentives - Self-consequences	Self-observation: - Metacognitive monitoring - Self-recording	Self-judgment: - Self-evaluation - Causal attribution	Self-reaction: - Self-satisfaction/affect - Adaptive/defensive
Winne and Hadwin (1998)	1. Definition of task		3. Studying tactics		4. Adaptations	
	Task conditions: - Resources - Instructional clues - Time - Socia context Cognitive conditions - Beliefs, dispositions, & styles - Motivational factors & orientations - Domain knowledge - Knowledge of task - Knowledge of study tactics & strategies		Control: - Operation(s): Primitive, acquired (tactics & strategies)		- External evaluations	
	2. Goals & plan(s): standards					
	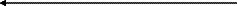		Monitoring: Cognitive evaluations		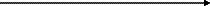	
Pintrich (2000)	1. Forethought, planning, and action		3. Monitoring	2. Control	4. Reaction & reflection	
			Cognition			
	- Target goal setting - Prior content knowledge activation - Metacognitive knowledge activation		- Metacognitive awareness and monitoring of cognition (feelings of knowing, judgments of learning)	- Selection and adaptation of cognitive strategies for learning, thinking	- Cognitive judgments - Attributions	
			Motivation			
	- Goal orientation adoption - Efficacy judgments - Ease of learning judgments - Perceptions of task difficulty - Task value activation - Interest activation		- Awareness and monitoring of motivation and affect	- Selection and adaptation of strategies for managing motivation and affect	- Affective reactions - Attributions	
			Behavior			
	- [Time and effort planning] - [Planning for self-observation of behavior]		- Awareness and monitoring of effort, time use, need for help - Self-observation of behavior	- Increase/decrease effort - Persist, give up - Help-seeking behavior	- Choice behavior	
			Context			
	- [Perceptions of task] - [Perceptions of context]		- Monitoring changing task and context conditions	- Change or renegotiate task - Change or leave context	- Evaluation of task - Evaluation of context	
Boekaerts (2006)	Work model: - (Meta) cognitive strategy use - Task-in-context - Motivational beliefs - Appraisal		Learning intention: growth pathway Affect: well-being pathway Volition			
Efklides (2011)			Metacognition and affect			
	- Metacognitive experiences (prospective) and metacognitive skills		- Metacognitive experiences (during) and metacognitive skills		- Metacognitive experiences (retrospective) and metacognitive skills	
			Cognition			
	- Task representation		- Cognitive processing		- Performance	
			Regulation of affect/effort			
	- Regulation of affect		- Regulation of effort		- Regulation of affect	
			Monitoring and control			
Miele and Scholer (2018)	Superordinate goal > task goal		Deep, Surface, divergent, convergent processing			
	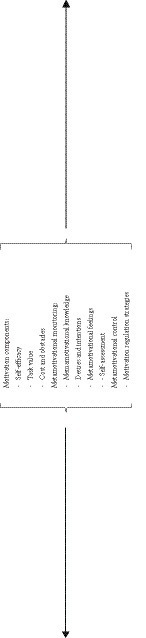					
Wirth et al. (2020)	Learning task requirements (long-term memory): - Search and retrieval		Learning performance (sensory memory): - Resonance Working memory: - Scheme construction - Organization - Elaboration - Rehearsal		Long-term memory - Store	

In the dynamic models, the components corresponding to metacognitive knowledge are located systematically in the phase prior to the start of task execution, except in the model of Miele and Scholer (2018). This model emphasizes that self-efficacy and the task value are modulated by metamotivational monitoring and control throughout the whole SRL cycle. The authors thus adopt a componential approach that we can qualify as “state-based,” complementary to the view of the components analysis in terms of traits, which prevail in the models.

The components related to metacognitive skills are linked to execution of the task, although in the model of Miele and Scholer (2018) motivation is controlled from the start to the end of the learning episode.

The components related to metacognitive experiences reflect the monitoring process that takes place during task execution. However, in the model of Miele and Scholer (2018), this process extends to the phase(s) prior to task execution, while those of Zimmerman and Moylan (2009) and Winne and Hadwin (1998) also explicitly includes self-evaluation after finalization of the task.

Finally, we can see that the dynamic models delimit SRL processes and states. However, the degree of detail varies depending on the metacognitive facets. Thus, metacognitive and metamotivational knowledge and beliefs are very detailed (particularly the latter). The learning strategies are generally referred to in a global way in the dynamic models, with the exceptions of the detailed cognitive strategies included in the models of Zimmerman and Moylan (2009) and Wirth et al. (2020), of resource management strategies in the model of Zimmerman (1998a) and of the motivational strategies in the model of Miele and Scholer (2018). Metacognitive experiences are also not detailed, although the model of Pintrich (2000) refers to metocognitive judgements and feelings, and the model of Miele and Scholer (2018) includes metamotivational feelings.

## 4 Discussion and conclusions

Our aim in the present study was to provide an up-to-date, comparative and integrative description of the major models of SRL proposed to date. Two research questions were posed. The first referred to delimiting the essential characteristics and components of the theoretical models on SRL. In this regard, we demonstrated that all of the models considered share a framework of ideas related to intellectual and affective-motivational functioning, interconnected in a prototypical recurring temporal sequence. However, each model provides a particular focus within the common framework, in a similar way to a camera scanning an unknown landscape with a zoom that enables visualization of the underlying ecosystem.

The model of Zimmerman (1998b, 2000), which adopts a distant focus, established the structural basis of the representation of the SRL construct: 3 basic process stages (before, during and after performance of a task/learning episode), in which essential processes and dimensions are located. Unsurprisingly, this is the model most frequently cited in the scientific literature. Boekaerts (1991, 2006) and also Winne and colleagues (Winne and Hadwin, 1998; Winne and Perry, 2000) adopted a more closely focused approach and directed attention to the role of experiences and representations generated in response to a task, which would give rise to the development of action-reaction loops, advances and backwards steps throughout the basic phases of self-regulation. Pintrich (2000) adjusted the zoom to an intermediate distance, considering the different areas of self-regulation (cognition, motivation/affect, behaviour and context), while still detailing processes and dimensions. As discordant feature of Pintrich’s model relative to the others, although he recognises the role that the monitoring the student carries out of their own action of learning, this is circumscribed to a phase concurrent to the control phase (selection, application and adaptation of learning strategies), both corresponding to the execution of the learning task.

Regarding delimitation of the SRL components, theoretical elaborations in the field of metacognition and information processing have been fundamental, generating a consistent list of notions of fundamental personal and situational processes and dimensions. However, the location of the different components in specific phases of the SRL cycle, as considered in some of the classic dynamic models, may be misleading. Although the weight of some components (such as monitoring or self-schemes) may vary between phases, these can be manifested throughout the SRL cycle and in the different feedback loops generated during execution of the task (Bakhtiar and Hadwin, 2021). This aspect is clearly reflected in the modern dynamic models.

We have been able to identify various components of a common organizational framework; however, we have observed that the components are unequally weighted. While the components related to metacognitive knowledge are usually detailed in the models, those related to skills and metacognitive experiences are referred to more globally. The literature on SRL includes investigations on specific categories of the components, which complement the list that we have extracted from the models considered. These studies must be considered in order to obtain an overall view of the complex framework of processed and dimensions involved in SRL. Thus, regarding metacognitive knowledge, we have available analytical studies on epistemic beliefs (Schommer-Aikins, 2004; Muis and Singh, 2018), on motivational beliefs (Eccles and Wigfield, 2002) and on contextual conditions (Ben-Eliyahu and Bernacki, 2015; De la Fuente-Arias, 2017). Regarding metacognitive skills, classifications of (meta) cognitive and affective-motivational learning strategies have been proposed (Dresel et al., 2015; Martínez-López et al., 2021). The microprocesses executed by students in response to complex tasks, making use of hypermedia environments, have also been explored (Winne, 2017). Finally, the studies by Efklides (2002, 2006) can be highlighted in regard to the analysis of metacognitive experiences.

Our second research question considered the evolution of theoretical models of SRL. In comparison with classic models, the modern models are characterized by a focus that is relatively close to the action of learning. Although their relationship to the classic models is evident, the recent models generally provide a more recognisable view of the complexity and multidimensionality of the processes involved in SRL. Affective-motivational regulation is also given the necessary prominence in these models, along with the role of contextual conditions and attitudes and routines shaped in the personal history of learning experiences.

In summary, SRL has appeared as a central topic in Educational Psychology in the past few decades, and a series of shared assumptions regarding the nature of the construct have since been consolidated and a legacy has been built consisting of the processes and dimensions involved. Without these achievements it would be difficult to account for the large number of studies conducted in the field of SRL. The models have inspired recent lines of study including detailed analysis of online SRL (during its course) (Rovers et al., 2019), its manifestation in diverse instructional formats (e.g., collaborative learning, virtual environments, etc.) (Winne, 2017; Hadwin et al., 2018) and the role of affective/motivational self-regulation (Wolters, 2003; Pekrun and Stephens, 2009).

Thus, appreciable advances have been made in bridging the gap between the abstractions represented in SRL models and the reality of the phenomenon under study, in itself complex, multidimensional and contextual. However, as already pointed out by Jakešová and Kalenda (2014) regarding the classic models, the precision of the causal mechanisms involved in SRL remains limited; on the other hand, we can assume that at least some parts of the mechanisms represented by the models are bidirectional. Thus, the following are proposed as priority lines of further research in the field of SRL: (1) modelling the dimensions and processes involved in SRL in more precise terms, taking into account possible reciprocal causalities; and (2) reviewing the empirical evidence to support or, where appropriate, question new, more detailed models. Regarding the educational applications of the present and future theoretical analyses of SRL, their potential for inspiring general guiding principles and enhancing the effectiveness of programmes aimed at providing training in learning skills should be highlighted.

## Author contributions

CT: Conceptualization, Formal analysis, Methodology, Project administration, Supervision, Writing – original draft. MEM: Investigation, Methodology, Visualization, Writing – review & editing. EV: Conceptualization, Investigation, Methodology, Writing – original draft. ZM-L: Methodology, Visualization, Writing – review & editing.

## Footnotes

^1^In this case, we use the term “objective” rather than “goal,” as we consider the former is more in keeping with the aims pursued in a task execution. We use the term “goal,” throughout the text, to refer to more general purposes.
